# The systemic immune-inflammation index in coronary heart disease: a narrative review of thromboinflammation, phenotype-dependent utility, and clinical translation

**DOI:** 10.3389/fcvm.2026.1778315

**Published:** 2026-04-28

**Authors:** Mengyao Liu, Jiaojiao Mao, Xinyi Wang, Kaili Wang, Henghe Wang

**Affiliations:** 1 Cardiology Department, First Teaching Hospital of Tianjin University of Traditional Chinese Medicine, Tianjin, China; 2 National Clinical Research Center for Chinese Medicine, Tianjin, China

**Keywords:** coronary heart disease, phenotype-dependent, risk stratification, systemic immune- inflammation index, thromboinflammation

## Abstract

Coronary heart disease (CHD) is essentially a thrombo-inflammatory disease, and its key pathological mechanism lies in an acute thrombotic storm driven by the interaction between platelets, neutrophils, and lymphocytes. Existing inflammatory markers, which reflect only a single dimension, struggle to accurately capture this complex network. The Systemic Immune-Inflammation Index (SII), calculated using the formula (platelets × neutrophils/Lymphocytes), integrates the three dimensions of thrombosis, inflammation, and immunity, providing a novel composite indicator for quantifying the thrombo-inflammatory burden. This article provides a review of the latest evidence regarding SII in the risk stratification of coronary heart disease. Existing studies indicate that the predictive utility of SII exhibits significant phenotype dependence: it peaks in acute coronary syndrome (ACS) and related complications, accurately reflecting the intensity of acute thromboinflammatory storms; whereas in chronic coronary syndrome (CCS), its utility is diminished, serving merely as a tool for monitoring background inflammation. This dual characteristic of “acute warning and chronic monitoring” stems from its pathological specificity-the SII molecule (P × N) directly reflects the synergistic amplification effect of platelet-neutrophil interactions and performs best in thrombus-driven events. Clinical evidence supports the establishment of a three-tiered threshold system: (1) Acute-phase high-risk threshold (>900–1400), used for predicting ACS major adverse cardiovascular events (MACE) and stratifying mortality risk; (2) Complication stratification thresholds (screening: 450–650; diagnosis: >1,000), to guide the identification and intervention of microvascular complications such as no-reperfusion phenomenon (NRP); (3) Chronic-phase monitoring thresholds (>650), indicating anatomical complexity rather than an independent prognostic factor. However, the clinical translation of SII remains limited: cut-off values show high heterogeneity (459–2,174), comparisons with the Systemic Inflammatory Response Index (SIRI) and the Pan-Inflammatory Value (PIV) remain controversial, and most existing evidence comes from observational studies. Future efforts should focus on establishing the mechanism-specificity of SII by validating its association with direct thrombotic and inflammatory markers such as neutrophil extracellular traps (NETs), developing phenotype-adaptive risk models, and conducting randomized controlled trials to verify whether SII-guided intensified anticoagulation or anti-inflammatory interventions (such as colchicine) can improve clinical outcomes. The transition from a risk stratification tool to an indicator for precision treatment decision-making represents the next critical step in the clinical application of SII.

## 1 Introduction

Coronary heart disease (CHD) remains one of the leading causes of morbidity and mortality worldwide, and its growing burden is placing an increasingly heavy strain on society and the economy (1–4). In recent years, thrombo-inflammation has emerged as a frontier research area in elucidating the pathophysiological mechanisms of atherosclerosis (5–7). A growing body of evidence suggests that the interaction between platelet-driven thrombogenesis and neutrophil-mediated inflammatory responses may constitute the core pathophysiological pathway driving the onset and progression of CHD (8–11). This deepening understanding has spurred clinical exploration of anti-inflammatory therapies. From the landmark CANTOS study (12) to colchicine trials such as LoDoCo2 and COLCOT (13, 14), anti-inflammatory therapy has made the leap from theory to clinical practice. However, this breakthrough has also raised new clinical questions: How can we accurately identify patients at risk of “residual inflammation”? Existing inflammatory biomarkers, such as C-reactive protein (CRP) and high-sensitivity C-reactive protein (hs-CRP), while capable of reflecting systemic inflammatory burden, struggle to capture the cellular interaction mechanisms underlying thrombotic inflammation. This leads to a disconnect between “pathological mechanisms and clinical phenotypes” and creates a dilemma in identifying “residual inflammatory risk.” These limitations have spurred the need for novel composite indicators that can comprehensively reflect the state of the immune-thrombotic network.

**Figure 1:**
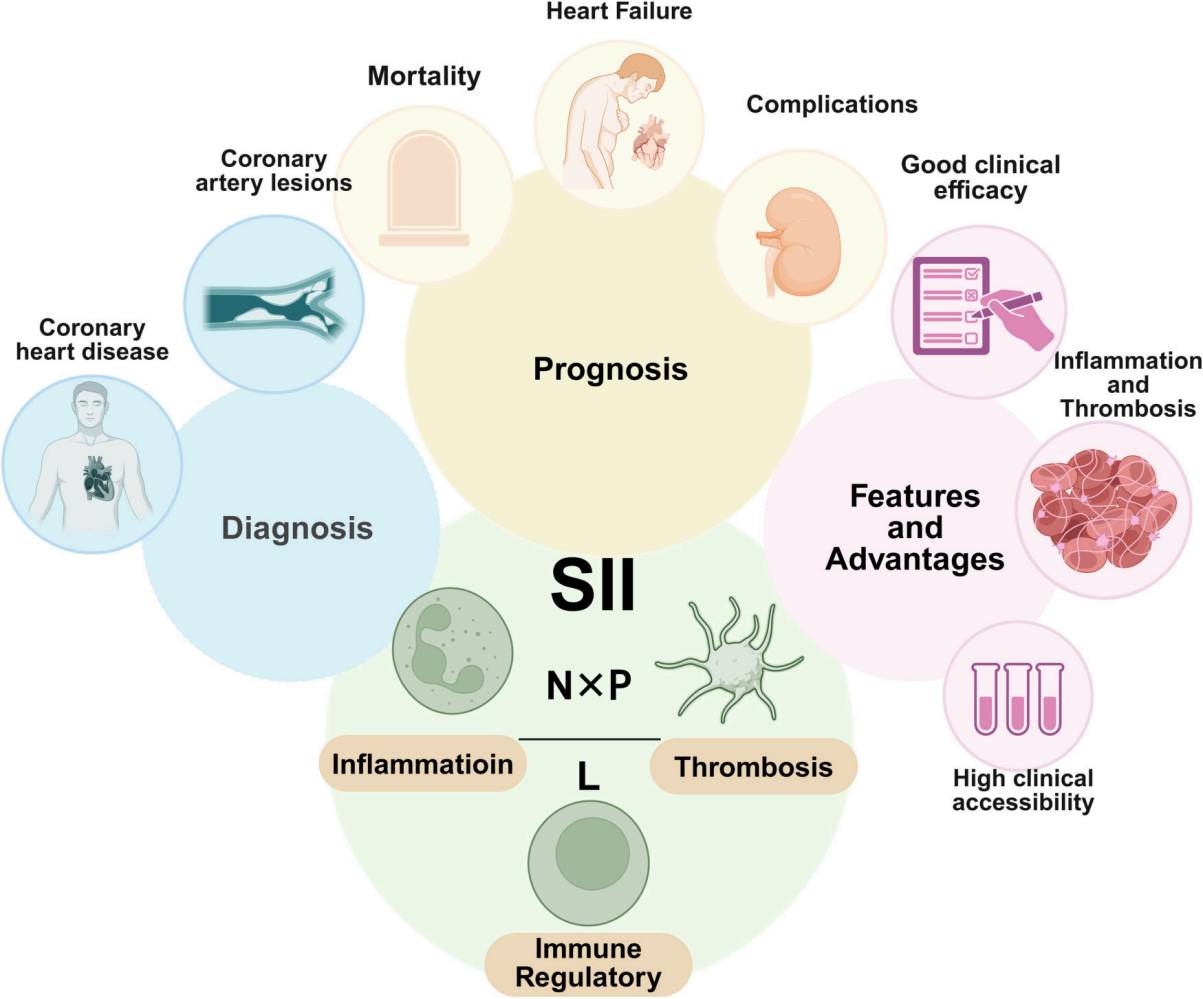
Predictive performance and clinical applications of SII in coronary heart disease. Created in BioRender. Liu, M. (2026). https://BioRender.com/wnp8hms.

The Systemic Immune-Inflammation Index (SII)-calculated as (neutrophil count × platelet count)/lymphocyte count-is a prime example of this paradigm shift: by capturing the relative abundance of blood cell subsets, it simultaneously captures the three-dimensional interactions among the immune, inflammatory, and thrombotic systems, aiming to quantify the dynamic state of the thrombosis-inflammation cycle in real time. Since its initial introduction in the field of oncology by Hu et al. in 2014 (15) and the subsequent validation of its value in cardiovascular prognosis assessment by Seo et al. in 2018 (16), the SII has accumulated substantial evidence in the prediction of coronary heart disease risk, severity assessment, and prognostic warning due to its broad clinical accessibility, cost-effectiveness, and comprehensive coverage of thrombotic-inflammatory pathophysiological dimensions (Figure 1). This review aims to systematically summarize the clinical application progress of SII in the aforementioned areas within the thrombosis-inflammation framework, critically evaluate its clinical applicability, discuss the limitations of existing studies, and propose future research directions.

## 2 Literature search and selection methods

This study conducted a systematic search of four major electronic databases-PubMed/MEDLINE, Embase, Web of Science, and the Cochrane Library-with a search cutoff date of December 2025. The search was restricted to English-language articles, with no restrictions on study design, population characteristics, or publication date, to ensure comprehensive coverage of the evolution of SII clinical applications.

In PubMed, a combined search strategy using MeSH terms and keywords was employed. MeSH terms included: “Coronary Artery Disease” [MeSH], “Acute Coronary Syndrome” [MeSH], “Myocardial Infarction” [MeSH], “Angina, Stable” [MeSH], and “Angina, Unstable” [MeSH]; Keywords included: “Systemic Immune-Inflammation Index,” “SII,” “systemic immune inflammation index,” “coronary heart disease,” “CHD,” “CAD,” “ACS,” “CCS,” “AMI,” “MI,” “STEMI,” and “NSTEMI.” Boolean operators were used to link disease and biomarker concepts with “AND” and synonyms within the same group with “OR.”

Similar search strategies were applied to Embase (using Emtree controlled vocabulary and keyword combinations), Web of Science (Core Collection), and Cochrane Library (Trials and Reviews). ScienceDirect was additionally searched as a full-text source to capture early-access articles. The specific syntax was adapted to each database's search interface while maintaining consistent concept coverage across all platforms.

An initial search yielded 446 relevant articles. After deduplication and screening of titles and abstracts, reviews, case reports, basic research studies, and irrelevant literature were excluded. Additionally, supplementary searches were conducted using backward citation tracking and ResearchRabbit's forward citation tracking to ensure that no important studies were overlooked. Following this process, a total of 151 clinical studies were ultimately included, covering clinical evidence regarding SII in the prediction of CHD risk, assessment of lesion severity, prognostic stratification, and warning of complications.

Given the narrative nature of this review, we did not follow the PRISMA guidelines for systematic reviews, nor did we employ strict double-blind screening or bias risk assessment tools; instead, we focused on a critical interpretation of evidence heterogeneity, integration of pathophysiological mechanisms, and clinical translational value. Original clinical studies (prospective/retrospective cohort, case-control, cross-sectional studies, and randomized controlled trials) served as the primary sources of evidence, while review articles were used solely to provide contextual support.

## 3 The pathophysiological basis of SII: a comprehensive interpretation within the thrombotic-inflammatory framework

### 3.1 The essence of the thrombotic-inflammatory mechanism in coronary heart disease

Atherosclerosis is essentially a thrombotic inflammatory disease (5–7). The pathological core of plaque progression to the terminal stage is not merely lipid deposition or mechanical stenosis, but rather a vicious positive feedback loop formed by platelet activation (thrombogenic arm), neutrophil recruitment (pro-inflammatory arm), and lymphocyte apoptosis (failure of immune surveillance)-this is the essence of “thrombosis-inflammation” (17). Activated platelets capture neutrophils via P-selectin, triggering their degranulation and the release of neutrophil extracellular traps (NETs); NETs further activate platelets and damage the endothelium, creating a self-amplifying thrombotic storm. Concurrently, a sharp decline in lymphocytes signals waning inflammation and the failure of tissue repair functions. It is the spatiotemporal specificity of these cellular-level interactions—rather than a simple increase in inflammatory molecule concentrations-that determines the clinical phenotype and prognosis of coronary heart disease.

### 3.2 SII's quantitative mapping of the thrombotic-inflammatory circuit

**Figure 2:**
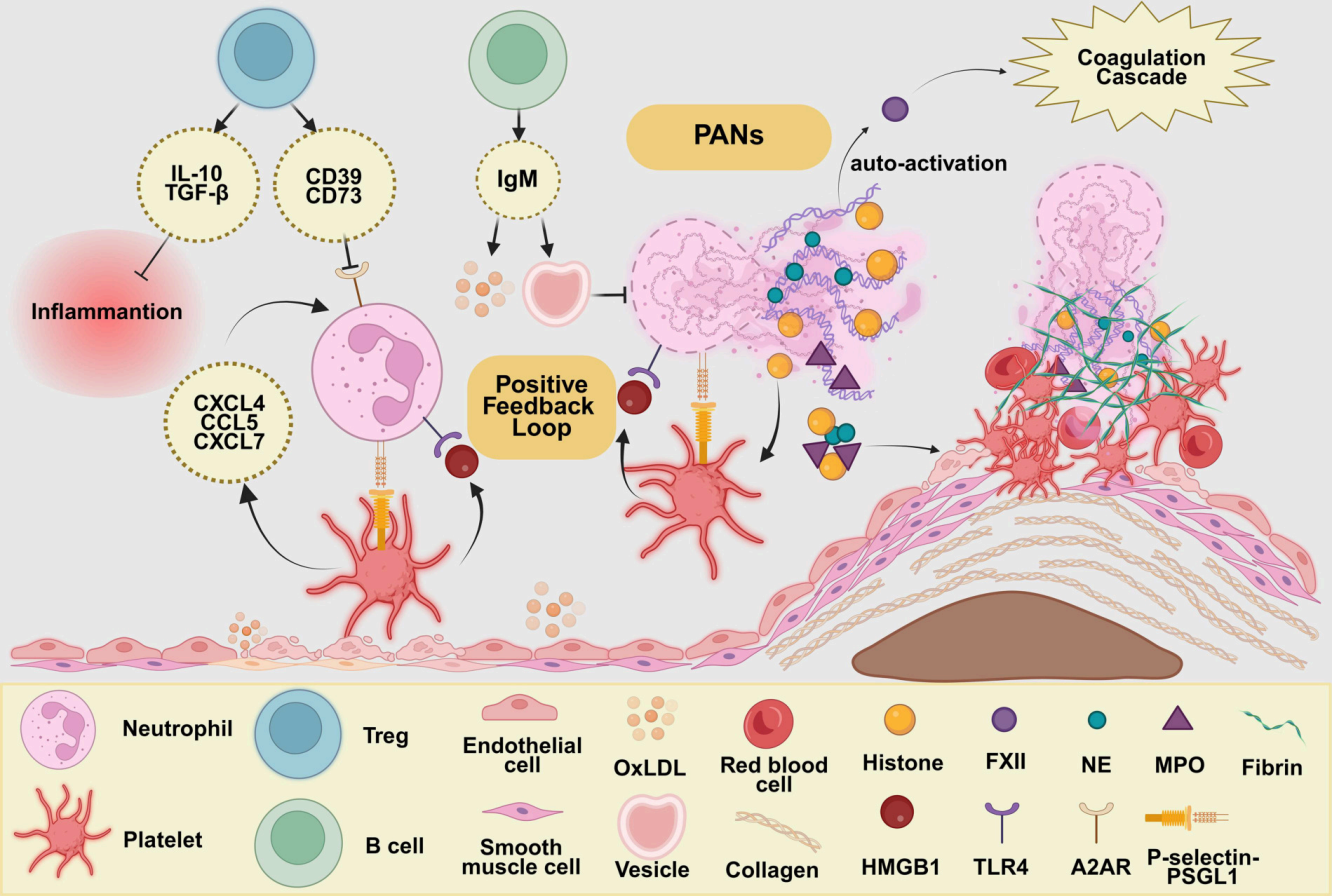
Schematic diagram of the SII thrombotic-inflammatory pathway. Created in BioRender. Liu, M. (2026). https://BioRender.com/hx3fb6s. The schematic is organized into three functional regions. The lower-left region illustrates platelets as “amplifiers” of inflammation: following endothelial damage activated platelets release chemokines and recruit neutrophils via P-selectin-PSGL1, thereby inducing NETs formation; PNAs anchor to the plaque site, amplifying the coagulation response. HMGB1 released by platelets further induces NETs release via TLR4, while NETs in turn reactivate platelets, forming a positive feedback loop. The central-right region depicts NETs as “triggers” of thrombotic inflammation: activated neutrophils release DNA, histones, and proteases to form NETs, which provide a scaffold for thrombi; NETs degrade collagen, disrupt the fibrin cap, activate platelets and coagulation factor XII, and inhibit protein C activation, thereby disrupting the anticoagulant balance and ultimately promoting acute arterial occlusion. The upper region shows lymphocytopenia as a “disruptive factor”: Tregs suppress platelet and NET activation via the CD39/CD73-adenosine-A2AR pathway and secrete IL-10/TGF-β to inhibit inflammatory responses; B cells secrete IgM to clear oxLDL and inhibit NET formation. Lymphopenia leads to the failure of immune regulation, causing the NET-platelet positive feedback loop to spiral out of control, thereby accelerating the progression of atherosclerosis.

The SII is calculated as N (neutrophil count)  ×  P (platelet count)/L (lymphocyte count). Using these three readily available hematological parameters, the SII directly quantifies the three key functional nodes in the aforementioned thrombotic-inflammatory loop. The numerator of the SII (N × P) reflects the combined effect of prothrombotic and pro-inflammatory drivers, while the denominator (L) represents endogenous immune regulatory capacity. (See Figure 2 for details.) The pathophysiological basis of each component of SII is detailed below.

#### 3.2.1 NETs act as “igniters” of thrombotic inflammation

Activated neutrophils release DNA, histones (such as H3/H4), myeloperoxidase (MPO), and neutrophil elastase (NE) to form NETs. These structures physically trap platelets, red blood cells, and procoagulants, providing a “scaffold” for thrombus formation (18). Proteases (such as NE) and histones within NETs damage smooth muscle cells and degrade collagen (19), accelerating the thinning and rupture of the fibrin cap and inducing thrombus formation; simultaneously, NETs directly activate platelets and promote platelet aggregation via cytoplasmic protease G and histones (20), NETs activate the intrinsic coagulation cascade through the negatively charged surface provided by their DNA scaffold, promoting the autoactivation of coagulation factor XII (FXII) (21). Furthermore, histones in NETs bind to and inhibit thrombin-regulated protein C activation (22), disrupting the anticoagulant balance and ultimately leading to acute occlusion of the arterial lumen (23, 24).

#### 3.2.2 Platelets act as “amplifiers” of inflammation

Following endothelial injury, platelets are the first to adhere and become activated. Activated platelets release soluble mediators, such as chemokines (including CCL5, CXCL4, and CXCL7), and, through adhesion molecules, such as P-selectin-P-Selectin Glycoprotein Ligand-1 (PSGL1), participate in the recruitment and activation of neutrophils and induce the formation of NETs (25–27). More importantly, platelets and neutrophils form stable platelet-neutrophil aggregates (PNAs) that anchor at plaque and thrombus sites, amplifying the coagulation response (8) and exacerbating plaque instability. High-mobility group box 1 (HMGB1) released by platelets further induces NETs release via Toll-like receptor 4 (TLR4) (28), while NETs activate platelets via histones, forming a positive feedback loop.

#### 3.2.3 Lymphocytopenia as a “regulatory factor”

Regulatory T cells (Tregs) hydrolyze extracellular ATP into adenosine via high expression of CD39/CD73; adenosine acts on the A2A adenosine receptor (A2AR) on the surface of platelets and neutrophils, significantly inhibiting platelet activation, adhesion, and NET release (29, 30). Tregs secrete Interleukin-10 (IL-10) and Transforming Growth Factor-beta (TGF-β) to suppress inflammatory responses in macrophages and dendritic cells (31, 32). B cells exert anti-atherosclerotic effects by secreting native IgM antibodies to clear oxidized low-density lipoprotein (oxLDL), thereby reducing macrophage uptake of oxLDL and the formation of foam cells, and by blocking vesicle-induced NETs, which in turn reduces the activation of the coagulation cascade (33–36). Lymphopenia impairs immune regulatory functions, promotes endothelial dysfunction and platelet activation, and, together with NET- and platelet-driven thromboinflammatory processes, contributes to a pathological imbalance that accelerates the progression of atherosclerosis (37).

In summary, SII can be understood as a mathematical expression of the thrombo-inflammatory burden: the numerator (N × P) represents the combined effect of prothrombotic and pro-inflammatory drivers, reflecting the intensity of the positive feedback loop between platelets and neutrophils; the denominator (L) represents endogenous immune regulatory capacity. Elevated SII indicates a loss of control in the thrombotic-inflammatory loop—specifically, the combined effects of platelet-driven thrombogenesis, neutrophil-mediated NET-induced inflammation, and immune imbalance caused by lymphocyte exhaustion. Therefore, SII is not only a statistically significant predictive indicator but also a direct reflection of the pathological essence of thrombosis and inflammation, making it a comprehensive biological biomarker that bridges the pathological mechanisms of coronary heart disease with clinical risk stratification.

## 4 Predictive performance of SII across different clinical phenotypes: a stratified analysis based on AUC and cutoff values

Clinical evidence indicates that the predictive performance of SII exhibits a distinct dependence on the pathological phase: it peaks during the acute phase of acute coronary syndrome (ACS) (the thromboinflammatory storm phase) (mean AUC 0.79) and declines significantly during the chronic phase of chronic coronary syndrome (CCS) (the low-grade background inflammation phase) [mean area under the curve (AUC) 0.61]. This “phenotype-dependent” pattern confirms the essence of SII-it specifically quantifies the acute pathological storm driven by platelet-neutrophil-lymphocyte interactions.

### 4.1 Acute coronary syndrome: precise quantification of the thrombotic inflammatory storm

The pathophysiological core of ACS is the acute thromboinflammatory response triggered by plaque rupture—explosive platelet activation provides a procoagulant surface (P), neutrophil infiltration releases NETs to form a thrombotic scaffold (N), and rapid lymphocyte apoptosis signals repair failure (L). The P × N/L formula proposed by SII precisely quantifies this three-dimensional interaction.

#### 4.1.1 Assessment of coronary lesions

The SII performed well in assessing the severity of coronary lesions in ACS patients. The SII had a mean AUC of 0.74 and a mean cutoff value of 825. See Tables 1, 2 for specific details.

**Table 1:** Researches on the relationship between the SII and the risk of coronary heart disease/coronary artery lesions.

Research Outcomes	Author	Research Type	Study Population	Optimal Cutoff Value	Research Findings
Risk of CHD	Süygün et al. (82)	Retrospective study	SAP	528.3	SII is a predictor of functional myocardial ischemia
Coronary Artery Lesions	Candemir et al. (75)	Retrospective study	SAP	750	SII is an independent predictor of high SxS (AUC 0.95)
	Altunova et al. (38)	Retrospective study	Patients with STEMI undergoing PCI	1,025.1	SII is an independent predictor of high RSS (AUC 0.82)
	Liu et al. (76)	Retrospective study	CCS	652.8	SII is an independent risk factor for the severity of coronary artery stenosis, with predictive performance superior to NLR, PLR, and CRP (AUC 0.86, 0.83, 0.73, and 0.80)
	Erdoğan et al. (77)	Retrospective, observational study	CCS	620	SII outperforms PLR and NLR in predicting significant coronary artery stenosis in patients with CCS (AUC 0.74, 0.69, and 0.66)
	Zhu et al. (42)	Retrospective study	STEMI	914	SII outperforms NLR and PLR in predicting the infarct size in STEMI patients (with AUC values of 0.59, 0.58, and 0.53 for SII, NLR, and PLR, respectively)
	Ji et al. (39)	Retrospective study	NSTEMI	837.5	SII is an independent risk factor for triple-vessel disease in patients with NSTEMI (AUC 0.80)
	Kelesoglu et al. (79)	Retrospective study	Patients with stable coronary artery disease presenting with stenosis of 95% or greater in a major coronary artery	729.8	SII is an independent predictor of poor CCC formation in stable coronary artery disease patients, with predictive performance superior to NLR and CRP (AUC 0.83, 0.78, and 0.69)
	Ji et al. (40)	Retrospective study	ACS	802.9	SII is a predictor of plaque fragility in ACS patients (AUC 0.77)
	Adali et al. (78)	Cross-sectional study	CTO	679.9	High SII levels are significantly associated with CCC deficiency (AUC 0.73)
	Yilu et al. (41)	Retrospective study	ACS	543.4	SII is superior to SIRI and PIV in predicting the progression of NCL in patients with ACS (AUC 0.73, 0.70, and 0.68)

**Table 2:** Summary of the predictive performance of SII across different clinical phenotypes of coronary heart disease.

Applications	Clinical phenotype	Clinical endpoint	Optimal Cutoff Value	AUC	Sensitivity/Specificity	References
Risk of CHD	SAP	Functional myocardial ischemia	528.27	0.59	48.5%/68.6%	(82)
Coronary Artery Lesions	STEMI	High RSS	1,025.1	0.82	73.8%/72.3%	(38)
	STEMI	Infarct size by CMR	914	0.59	62.6%/53.4%	(42)
	NSTEMI	Three-Vessel Lesions	837.5	0.80	66.9%/80.0%	(39)
	ACS	Plaque fragility	802.9	0.77	67.5%/79.6%	(40)
	ACS	NCL progression	543.4	0.73	67.2%/72.2%	(41)
	SAP	High SxS	750	0.95	86.2%/87.3%	(75)
	CCS	The severity of coronary artery stenosis	652.8	0.86	71.0%/86.0%	(76)
	CCS	Significant coronary artery stenosis	620	0.74	78.4%/64.0%	(77)
	CCS	Poor CCC	729.8	0.83	78.4%/74.6%	(79)
	CTO	Poor CCC	679.9	0.73	74.5%/43.2%	(78)
Prognosis and Risk Stratification	STEMI	MACE	1,915.8^a^/696.4^b^	0.90^a^/0.89^b^	89.7%/81.4%^a^ 76.7%/88.0%^b^	(45)
	STEMI	1-year MACE	/	0.84	63.8%/88.0%	(47)
	STEMI	MACE	951.7	0.74	64.6%/73.6%	(49)
	STEMI	30-day mortality	1,036	0.71	70%/59%	(52)
	ACS	MACE	955.8	0.88	79.5%/87.9%	(48)
	ACS	MACE	713.9	0.71	63.6%/71.2%	(50)
	ACS	30-day MACE	866	0.87	74.2%/79.0%	(46)
	AMI	MACE	1,085.6	0.68	56.8%/74.2%	(44)
	Elderly patients with NSTEMI	In-hospital mortality and long-term mortality	2,174	0.86	80.0%/85.4%	(53)
	CTO	MACE	572.8	0.63	/	(80)
	CCS	MACE	694.3	0.59	/	(81)
	AMI	Early apical left ventricular thrombus formation	579	0.74	75.8%/72.8%	(58)
	MI	ST	636	0.74	55.6%/76.7%	(59)
	NSTEMI	Significant coronary thrombus burden	1,103	0.79	74.4%/74.6%	(60)
	STEMI	NRP	/	0.79	/	(64)
	STEMI	NRP	1,028	0.84	79%/70%	(63)
	STEMI	NRP	458.9	0.64	94.1%/28.9%	(43)
	ACS	NRP	613	0.80	84%/66%	(62)
	ACS	NRP	975	0.84	74%/80%	(65)
	STEMI	CIN	1,136	0.67	74%/55%	(70)
	STEMI	CIN	1,282	0.83	76.1%/86.7%	(67)
	NSTEMI	CIN	709	0.78	74%/74%	(68)
	NSTEMI	CIN	933.2	0.79	77.6%/69.2%	(69)

a 12 h after baseline.

b 1 month after baseline.

In STEMI patients, the AUC for SII in predicting a high residual SYNTAX score (RSS) reached 0.82 (cutoff value 1025) (38); the AUC for predicting three-vessel disease in non-ST-elevation myocardial infarction (NSTEMI) patients was 0.80 (cutoff value 838) (39). Furthermore, SII has predictive value for plaque vulnerability (AUC 0.77, cutoff 803) (40) and non-target lesion (NCL) progression after percutaneous coronary intervention (PCI) (AUC 0.73, cutoff 543) (41), with superior performance compared to the Systemic Inflammatory Response Index (SIRI) and the Pan-Inflammatory Value (PIV).

The pathophysiological basis for this association lies in the fact that diffuse coronary artery disease is a structural consequence of systemic thromboinflammatory activity. A high SII indicates platelet-neutrophil synergistic activation across multiple vascular beds, which anatomically manifests as multifocal, diffuse lesions and poor collateral circulation. It is worth noting that SII outperforms Neutrophil-to-Lymphocyte Ratio (NLR) and Platelet-to-Lymphocyte Ratio (PLR) in assessing the extent of myocardial infarction (42), further confirming that it reflects the thrombotic-inflammatory-driven tissue injury burden rather than mere anatomical stenosis.

However, the association between SII and the static SYNTAX score (SxS) is not always significant. Some studies have shown no correlation between SII and the TIMI flow classification (43), which may be because SxS reflects long-term anatomical accumulation, whereas SII quantifies the acute thromboinflammatory burden; thus, their pathological bases do not fully align. Furthermore, routine antiplatelet therapy in STEMI patients may suppress the platelet component of SII. This, in turn, suggests that SII has greater predictive value for acute complications than for the assessment of static anatomy.

#### 4.1.2 Prognostic risk stratification and dynamic monitoring

SII demonstrates excellent predictive performance for major adverse cardiovascular events (MACE) in ACS patients (44–57) (mean AUC 0.79, mean cutoff value 1178), significantly outperforming NLR and PLR (49, 50, 56). Specifically, SII measured 12 h after baseline demonstrated the best predictive ability for in-hospital MACE in STEMI patients (AUC 0.90, cutoff value 1916) (45), with AUCs of 0.87 (46) and 0.84 (47) for predicting MACE at 30 days and 1 year after PCI, respectively. See Tables 2, 3 for specific details.

**Table 3:** Researches on the relationship between SII and the risk of major adverse cardiovascular events in coronary heart disease.

Research Outcomes	Author	Research Type	Study Population	Optimal Cutoff Value	Research Findings
The composite endpoint of adverse cardiovascular events	Saylik and Akbulut (49)	Retrospective, observational study	STEMI	951.7	SII is an independent predictor of MACE in STEMI patients, with predictive capability superior to NLR and PLR (AUC 0.74, 0.68, and 0.61)
	Karadeniz et al. (48)	Cross-sectional study	ACS	955.8	SII is an independent predictor of adverse outcomes in ACS patients, with predictive efficacy surpassing that of PLR and NLR (AUC 0.88, 0.67, and 0.77)
	Wei et al. (44)	Retrospective study	AMI	1,085.6	SII has predictive value for MACE occurrence in AMI patients (AUC 0.68)
	Li et al. (56)	Retrospective study	AMI	970	SII has greater predictive value for in-hospital MACE in AMI patients than PLR and NLR (AUCs of 0.684, 0.597, and 0.654, respectively)
	Gao et al. (50)	Retrospective study	ACS	713.9	SII is a significant predictor of MACE risk in ACS patients, with greater predictive power than NLR and PLR (AUCs of 0.71, 0.68, and 0.56, respectively).
	Liu et al. (45)	Retrospective study	STEMI	1,915.77/696.43	SII at 12 h and 1 month from baseline is an independent predictor of in-hospital and out-of-hospital MACE (AUC 0.90, and 0.89)
	Zhao et al. (80)	Retrospective study	CTO	572.8	SII is an independent predictor of MACE, demonstrating superior predictive performance compared to NLR, PLR, and hs-CRP (AUC 0.63, 0.60, 0.62, and 0.58)
	Yang et al. (81)	Retrospective study	CAD patients undergoing PCI	694.3	Elevated SII is significantly associated with increased risk of cardiac death, non-fatal MI, non-fatal stroke, MACE, and total major events (AUC 0.59)
	Wang et al. (57)	Retrospective, observational study	STEMI	/	Elevated SII is associated with an increased risk of in-hospital HF
	Cui et al. (51)	Retrospective study	MI	770	SII is an independent predictor of 28-day mortality in patients with MI
	Vatan et al. (52)	Retrospective study	STEMI patients undergoing PCI	1,036	Elevated SII levels are independently associated with 30-day mortality following PCI in patients with STEMI (AUC 0.71)
	Yaşan et al. (54)	Observational study	NSTEMI	/	SII is positively correlated with long-term mortality in NSTEMI patients
	Orhan et al. (53)	Retrospective study	Elderly patients with NSTEMI	2,174	SII is an independent predictor of in-hospital mortality and long-term mortality in elderly patients with NSTEMI (AUC 0.86)
	Cai et al. (55)	Retrospective study	CHD	/	In a comprehensive assessment integrating the TyG index with multiple hematological inflammatory markers (including SII, SIRI, NLR, and PLR), SII emerged as the most significant contributor in predicting cardiovascular mortality among CHD patients
	Zhang et al. (47)	Retrospective study	STEMI patients undergoing PCI	/	Higher SII is independently associated with 1-year MACE in STEMI patients undergoing PCI (AUC 0.84)
	Babes et al. (46)	Retrospective study	ACS	866	Elevated SII predicts the risk of 30-day MACE occurrence in ACS patients, demonstrating superior predictive performance to NLR and CRP in STEMI (AUC 0.87, 0.70, and 0.69)
	Ren and Liu (37)	Retrospective study	Patients with CHD and HF	589.31	SII is a predictor of MACE risk in patients with CHD and HF (AUC 0.79)
	Wang et al. (104)	Retrospective study	STEMI patients undergoing PCI	/	Both SII and GRACE scores are independent predictors of short-term MACE occurrence, and their combined predictive efficacy surpasses that of either single indicator
	Zhu et al. (105)	Retrospective study	STEMI	/	SII and NT-proBNP are independent predictors of long-term MACE in STEMI patients, and their combined predictive performance surpasses that of either marker alone (The AUC for SII was 0.65, the AUC for NT-proBNP was 0.77, and the AUC for the combination of SII and NT-proBNP was 0.84)

The value of dynamic monitoring is a unique advantage of SII during the acute phase. Studies have found that elevated SII levels in the late phase carry greater prognostic significance than early elevations (47), and persistent elevations indicate prolonged inflammation and poor short-term prognosis (46). A large cohort study (*n* = 1,103) showed that patients with SII >956 had a 48.7-fold increased risk of MACE (AUC 0.88) (48). SII also demonstrates robust performance in predicting mortality risk (51–55), particularly in elderly patients with NSTEMI (AUC 0.86, cutoff value 2,174) (53). Furthermore, SII has been confirmed as the most important predictor of cardiovascular mortality (contribution rate 34.4%) (55).

The high predictive performance in the acute phase (AUC >0.80) stems from SII's comprehensive capture of the three-dimensional interactions of the thrombotic inflammatory storm-explosive platelet activation directly drives microvascular occlusion, neutrophil infiltration mediates myocardial reperfusion injury, and rapid lymphocyte apoptosis foreshadows impaired repair function. This “superior performance” has clear pathological correlates, whereas the efficacy of NLR and PLR is limited due to the absence of key dimensions (platelet or neutrophil synergy).

#### 4.1.3 Complication prediction

The SII demonstrates consistent predictive performance for acute complications of ACS (mean AUC 0.77, mean cutoff value 859), exhibiting a unified predictive capability across anatomical levels—from macroscopic intraventricular thrombi to microscopic microcirculatory emboli. See Tables 3, 4 for specific details.

**Table 4:** Researches on the relationship between SII and the risk of complications associated with coronary heart disease.

Research Outcomes		Author	Research Type	Study Population	Optimal Cutoff Value	Research Findings
CHD-Related Complications	Thrombotic events	Tok et al. (58)	Retrospective, observational study	Patients with anterior wall AMI	579	SII is a independent predictor of early apical left ventricular thrombus formation (AUC 0.74)
		Akboga et al. (61)	Retrospective study	ACS patients undergoing PCI	/	SII is an independent predictor of acute ST
		Zheng et al. (59)	Retrospective study	Patients with MI undergoing PCI	636	SII is an independent risk factor for ST (AUC 0.74)
		Özkan et al. (60)	Retrospective study	NSTEMI patients who underwent PCI	1,103	SII is an independent predictor of significant coronary thrombus burden in patients with NSTEMI (AUC 0.79)
	NRP	Çelik et al. (64)	Retrospective study	STEMI	/	SII is independently associated with NRP and demonstrates superior predictive performance compared to NLR and PLR (AUC 0.79, 0.77, and 0.76)
		Cakmak et al. (62)	Cross-sectional study	ACS	613	Elevated SII levels are independently associated with NRP in ACS patients undergoing CABG (AUC 0.80)
		Esenboğa et al. (63)	Retrospective study	Patients undergoing primary PCI for STEMI	1,028	SII is independently associated with NRP in patients with STEMI primary undergoing initial PCI (AUC 0.84)
		Özen et al. (65)	Retrospective study	Patients with ACS undergoing CABG/PCI	975	SII is an independent predictor of NRP in ACS patients undergoing CABG/PCI (AUC 0.84)
		Rostami et al. (43)	Prospective cohort study	STEMI	458.9	SII can predict the NRP in STEMI patients undergoing initial PCI (AUC 0.64)
	CIN	Öztürk et al. (70)	Retrospective study	Patients undergoing primary PCI for STEMI	1,136	SII outperforms PLR, NLR, and CRP in predicting CIN in STEMI patients undergoing primary PCI (AUC 0.67, 0.58, 0.56, and 0.56)
		Karauzum et al. (67)	Retrospective study	Patients undergoing primary PCI for STEMI	1,282	SII demonstrates superior predictive capability for CIN occurrence in STEMI patients undergoing primary PCI (AUC 0.83)
		Tezen et al. (68)	Cross-sectional study	NSTEMI	709	SII is an independent predictor of CIN in NSTEMI patients (AUC 0.78)
		Kelesoglu et al. (69)	Retrospective study	NSTEMI	933.2	The level of SII at admission is independently associated with the development of CIN after PCI in patients with NSTEMI (AUC 0.79)
		Ma et al. (72)	Retrospective study	STEMI	653.7	Elevated SII and hs-CRP levels are risk factors for CIN in STEMI patients, and their combined use demonstrates superior predictive performance compared to either marker alone (AUC for SII: 0.76; AUC for hs-CRP: 0.77; AUC for the combined SII-hsCRP model: 0.83)
		Shen et al. (71)	Retrospective study	STEMI patients undergoing emergency PCI	1,085.0	The combined use of SII and NT-proBNP demonstrates superior efficacy compared to either marker alone (AUC for SII: 0.65, AUC for NT-proBNP: 0.66, AUC for the combined SII and NT-proBNP model: 0.73)
		Zhu et al. (73)	Retrospective study	ACS patients undergoing PCI	736.1	Both SII and CHA2DS2-VASC scores are independent risk factors for CIN, and their combined use improves predictive accuracy (AUC for SII: 0.69; AUC for the CHA2DS2-VASC score: 0.80; AUC for the combined SII and CHA2DS2-VASC score model: 0.83)

First, regarding the prediction of thrombotic events, there is a high degree of consistency in the prediction of left ventricular thrombus (LVT; AUC 0.74, cutoff value 579), stent thrombosis (ST; AUC 0.74, cutoff value 636), and significant coronary thrombus burden (AUC 0.79, cutoff value 1103) (58–61). All three share the pathophysiological basis of “platelet-neutrophil synergistic activation”: platelets provide procoagulant phospholipid surfaces, neutrophils release NETs to stabilize thrombus structure, and lymphocyte depletion lifts the inhibitory effect of inflammation. The P × N component of SII directly reflects this mechanism, enabling it to identify systemic prothrombotic tendencies regardless of whether thrombi ultimately form in the cardiac chambers, stents, or the main coronary arteries.

Second, regarding the prediction of microcirculatory dysfunction. The ability to predict no-reperfusion phenomenon (NRP) and contrast-induced nephropathy (CIN) further validates SII's capacity to go beyond macroscopic vascular lesions.

SII demonstrates excellent predictive performance for NRP (AUC 0.79–0.84) (62–65), but the optimal cutoff value varies depending on the clinical objective. For screening purposes, a low cutoff value (approximately 460) is recommended, yielding a sensitivity as high as 94.1% (43) and identifying the vast majority of potential NRP patients; for diagnosis and to reduce overtreatment, a high cutoff value (>1,000) (63), which significantly improves specificity, effectively rules out false positives, and avoids unnecessary intensive anticoagulation or mechanical protection in low-risk patients. This “low-threshold screening–high-threshold confirmation” stratification strategy allows SII to flexibly adapt to various clinical scenarios, ranging from early identification to precise intervention.

Prediction of CIN shows heterogeneity (AUC 0.67–0.83) (62, 66–69). Most studies indicate that SII has high predictive performance for CIN in STEMI patients, with an AUC as high as 0.83 (67); in some cases, it outperforms NLR, PLR, and CRP (67, 70), and offers synergistic value when combined with hs-CRP, N-terminal pro-brain natriuretic peptide (NT-proBNP), and the CHA₂DS₂-VASc score (71–73). However, some studies have found that SIRI and neutrophil-to-albumin ratio (NPAR) outperform SII in predicting CIN (74), which may reflect the heterogeneity of CIN's pathophysiological mechanisms: when CIN is primarily driven by simple ischemia-reperfusion and oxidative stress in the renal medulla, SIRI-which includes monocytes-or NPAR-which reflects acute-phase proteins-may be more sensitive; whereas when CIN is accompanied by significant microthrombosis (e.g., PNAs embolism), SII retains high predictive performance (AUC up to 0.83). This suggests that the predictive value of SII is mechanism-dependent-it performs best in complications dominated by thrombotic components (ST, NRP, thrombotic CIN), while its efficacy is relatively limited in injuries dominated by pure ischemia or oxidative stress.

Although NRP and CIN present with different clinical manifestations, both share a microcirculatory thrombotic-inflammatory pathway-NRP reflects embolism and spasm in myocardial microvessels, while certain CIN phenotypes (particularly those accompanied by microthrombotic embolism) reflect thrombotic ischemic-inflammatory injury in renal medullary microvessels. By quantifying the microthrombotic response (platelet count reflects microthrombotic burden, neutrophils reflect the intensity of inflammatory infiltration, and lymphocytopenia indicates impaired endothelial repair), SII serves as a comprehensive assessment tool for the systemic microcirculatory status following PCI.

The consistent cross-level predictive performance of SII regarding LVT, ST, coronary thrombus burden, and microcirculatory complications confirms that this cross-organ, cross-level (macrovascular, device-related, microembolism) quantitative capability makes SII a comprehensive assessment tool for systemic microcirculatory status after PCI. Patients with high SII scores may benefit from enhanced pre-treatment antiplatelet and anti-inflammatory therapy as well as mechanical thrombus protection, thereby paving the way for the establishment of a unified risk assessment framework.

### 4.2 Chronic coronary syndrome: mild monitoring of persistent low-grade inflammation

In CCS, the predictive power of SII is significantly reduced. Pathology at this stage is characterized primarily by persistent low-grade inflammation, reflecting a shift in SII's function from a “storm monitor” to a “background inflammation gauge.”

#### 4.2.1 The “paradox” of lesion assessment and its interpretation

The performance of SII in assessing coronary lesions in patients with CCS appears to be acceptable (mean AUC 0.82, mean cutoff value 687), but significant heterogeneity is present. In patients with stable angina pectoris (SAP), the AUC for SII in predicting a high SxS (≥32) reached 0.95 (cutoff value 750) (75), the AUC for predicting severe coronary stenosis was 0.86 (cutoff value 653) (76), and the AUC for predicting fractional flow reserve (FFR) scores ≤0.80 was 0.74 (cutoff value 620) (77); The AUCs for predicting poor coronary collateral circulation (CCC) were 0.73 and 0.83, with cutoff values of 679.9 (78) and 729.8 (79), respectively. See Tables 1, 2 for specific details.

This “high performance” must be viewed dialectically. The elevated SII scores in CCS patients (mean cutoff of 687, approximately 42% lower than the 1178 observed in ACS) reflect the structural consequences of long-term inflammatory accumulation—diffuse lesions, calcification, and poor collateral circulation are the products of persistent chronic low-grade thromboinflammatory activity. At this stage, SII does not predict “acute events” but rather indicates the biological activity of the lesions (vulnerable plaque burden). It is worth noting that the high predictive value of SxS in SAP patients (AUC 0.95) may be influenced by selection bias and should be interpreted with caution.

#### 4.2.2 Decline in prognostic accuracy

The predictive accuracy of SII for MACE was significantly reduced in patients with CCS (mean AUC 0.61, mean cutoff value 634). In patients with Chronic Total Occlusion (CTO), the AUC for SII in predicting long-term MACE was only 0.63 (80); in the general CCS population, the AUC dropped to 0.59 (81). Furthermore, the AUC for SII in predicting functional myocardial ischemia in SAP patients was only 0.59 (cutoff value 528) (82), suggesting its limited value in predicting primary risk during the chronic phase. See Tables 2, 3 for details.

The chronic phase is characterized primarily by persistent low-grade inflammation, lacking the three-dimensional interactive amplification seen in acute thrombotic storms. At this stage, monocyte-macrophage-driven chronic inflammation predominates, and SII loses the pathological basis of platelet-neutrophil synergistic amplification, reflecting only the background inflammatory burden; consequently, its efficacy naturally diminishes.

### 4.3 Summary of the phenotype-dependent characteristics of SII

In summary, the predictive performance of SII exhibits marked “scenario-dependence”; the distribution of its AUC and cutoff values collectively reflects pathological differences in the intensity of the thromboinflammatory burden.

#### 4.3.1 Differences between the acute and chronic phases

In the acute phase (ACS), the AUC for SII in predicting MACE and complications is generally >0.80, with relatively high cutoff values (837–1,282, and slightly higher in STEMI patients than in NSTEMI patients), which aligns with the pathological intensity of the “thrombotic-inflammatory storm”.

In the chronic phase (CCS), the AUC decreased to 0.59–0.63, and the cutoff values dropped significantly (528–750), reflecting the background burden of persistent low-grade inflammation.

#### 4.3.2 Stratification characteristics by endpoint type

For process-related events (NRP, CIN, thrombus burden), SII demonstrated robust predictive ability for stability (AUC 0.67–0.84) with a concentrated cut-off range (613–1,136). These events are essentially a direct extension of the acute-phase thrombotic inflammatory process and align with the pathological basis of lesion burden.

For endpoint events (MACE), predictive performance exhibits a “bimodal” distribution: low thresholds (572–696) correspond to low performance (AUC 0.59–0.63), while high thresholds (800–2,174, particularly in the elderly population) correspond to high performance (AUC 0.86–0.90).

#### 4.3.3 Sources of heterogeneity in cutoff values

In addition to the pathological phase and type of endpoint, variability in cutoff values is also influenced by the treatment context: long-term antiplatelet therapy may suppress platelet signaling pathways, leading to systematically lower thresholds in the chronic phase compared to the acute phase. Furthermore, variations in sample size, follow-up duration, and endpoint definitions also contribute to heterogeneity.

In summary, SII should be regarded as a phase-specific marker of acute thromboinflammatory events-using a threshold of >1,000 for high-risk screening during the acute phase of ACS, employing thresholds of 450–650 and 1,000 before and after PCI for screening and confirming complications, respectively, and using a threshold of >650 to identify anatomical complexity during the chronic phase of CCS-rather than as an independent predictor of long-term prognosis. This precise “scenario-threshold” alignment is a clinical manifestation of the mechanism-specific nature of SII. Future research and clinical applications should appropriately define the value of SII based on specific clinical scenarios, avoiding a “one-size-fits-all” interpretation.

## 5 Advantages and clinical significance of SII compared to other inflammatory markers

By integrating platelets, neutrophils, and lymphocytes, SII bridges the gap between “single inflammatory molecules” and “thrombotic inflammatory networks.” Its advantages lie not only in improved predictive accuracy but also in its precise reflection of the pathological essence of coronary heart disease.

### 5.1 Comparison with classical acute-phase proteins: from downstream effects to cell-cell interactions

CRP, hs-CRP, and other liver-synthesized downstream products of inflammation (83, 84) are subject to multiple limitations, including a disconnect from underlying mechanisms, limited predictive value, and stability issues.

First, from a mechanistic perspective, CRP, hs-CRP, and similar markers merely reflect the terminal state of systemic inflammatory burden; they cannot distinguish between infectious inflammation and thrombotic inflammation, nor can they quantify the three-dimensional interactions among platelets, neutrophils, and lymphocytes. This is because, as acute-phase reactants, CRP has a weak association with coronary atherosclerotic burden and struggles to reflect plaque fragility or thrombotic potential. In contrast, SII directly corresponds to the three core components of the thrombotic-inflammatory loop, accurately depicting the vicious cycle of “inflammation driving thrombosis and thrombosis exacerbating inflammation.”

Second, regarding predictive performance, multiple studies have found that traditional acute-phase proteins have limited independent predictive ability (85, 86). Large-scale studies have confirmed that adding CRP to traditional risk models increases the C-statistic by only 0.0039 (87); a prospective study (ENTIRE-TIMI-23) even failed to find an independent association between CRP and mortality or recurrent myocardial infarction (88). Head-to-head comparisons show that SII outperforms CRP (70, 76, 79, 89) and hs-CRP (80) in predicting coronary artery lesions and complications. This advantage stems from the fact that SII captures cell-level thrombotic-inflammatory interactions, rather than downstream molecules synthesized by the liver.

Finally, regarding stability: CRP is susceptible to interference from non-cardiovascular factors such as infection, metabolic syndrome, and obesity (90); it also exhibits significant individual baseline variability (84, 91–93). In contrast, SII is based on routine blood tests and, by presenting data as a ratio, compensates for differences in absolute cell counts between individuals, making it more stable in reflecting the thromboinflammatory burden.

### 5.2 Comparison with traditional hematological markers: from two-dimensional ratios to three-dimensional integration

In the evolutionary spectrum of inflammatory markers, the NLR and PLR can be regarded as “first-generation” indicators marking the transition from single to composite markers, while SII represents a key leap from “two-dimensional” to “three-dimensional” analysis.

From a pathophysiological perspective, NLR and PLR cover only a single dimension of the thrombosis-inflammation network-NLR reflects the “inflammation-immune” axis, while PLR reflects the “thrombosis-immune” axis. Both lack critical synergistic links and are unable to quantify the synergistic amplification effect of the “thrombosis-inflammation” cycle. Platelets are not only central mediators of thrombosis but can also directly activate neutrophils by releasing mediators such as P-selectin, thereby driving the inflammatory response; in turn, the NETs released by activated neutrophils promote platelet aggregation. By integrating platelets and neutrophils, SII accurately captures the intensity of this vicious cycle. This also explains why SII demonstrates particularly outstanding predictive performance in acute thrombotic events, whereas the performance of NLR and PLR in such scenarios is relatively limited.

Multiple head-to-head studies have confirmed that the discriminatory ability of SII is significantly superior to that of NLR and PLR. In the assessment of coronary lesion severity, the AUC of SII for predicting a high SxS (≥32) reached 0.95, which was significantly higher than that of NLR (0.93) and PLR (0.88) (75); SII also performed better in the assessment of adverse outcomes based on the Gensini score, FFR, infarct size, and CCC (42, 76, 77, 79). In the prediction of MACE in STEMI patients, the AUC for SII was 0.74, whereas those for NLR and PLR were only 0.68 and 0.61, respectively, representing a significant difference (49). Furthermore, SII demonstrated superior discriminatory ability in predicting acute complications (such as NRP and CIN) (64, 67, 70).

### 5.3 Comparison with next-generation composite biomarkers: mechanistic differences and clinical context

Comparisons between SII and SIRI and PIV reveal context-dependent differences, which precisely reflect the dynamic weighting of the “thrombosis-inflammation” network across different pathological stages.

SII is characterized by a higher proportion of platelets and is particularly sensitive to “platelet-neutrophil interactions,” making it highly suitable for reflecting platelet activation and NET formation during acute thrombotic events. Consequently, SII excels in monitoring acute thrombotic storms; in scenarios such as the acute phase of ACS, ST, and NRP, platelet-neutrophil interaction (P × N) serves as the core driver of the pathological storm. In these cases, the predictive performance of SII (AUC 0.77–0.88) is generally superior to that of SIRI, as monocytes play a relatively minor role in acute thrombus formation. For example, in predicting non-target lesion progression after PCI, SII (AUC 0.73) outperforms SIRI (0.70) and PIV (0.68) (41).

In contrast, SIRI (neutrophil×monocyte/lymphocyte ratio) emphasizes the monocyte component and is more sensitive to “monocyte-macrophage-mediated chronic inflammation,” demonstrating a close association with the progression of atherosclerosis and plaque instability. Therefore, the advantage of SIRI lies in monitoring the accumulation of chronic lesions; it may perform better in predicting the severity of coronary artery lesions (such as SxS and diffuse lesions) and long-term MACE (94–100). This is because monocytes, as the core drivers of atherosclerotic progression (macrophage infiltration, formation of necrotic cores), carry greater pathological significance in chronic low-grade inflammation than platelets.

PIV (neutrophils×platelets×monocytes/lymphocytes) integrates these three inflammatory pathways, comprehensively covering both acute thrombotic inflammation and the burden of chronic atherosclerosis. PIV is positioned as a comprehensive immune assessment tool. Studies have found that the AUC values of PIV in predicting in-hospital outcomes for patients with STEMI, NSTEMI, and CCS are slightly higher than those of SII (101–103). This advantage may stem from PIV's integrative strengths and comprehensive assessment capabilities. However, the large number of parameters may also result in PIV lacking the acute-phase specificity of SII or the tissue specificity of SIRI.

In summary, these three markers do not simply represent a hierarchy of superiority or inferiority; rather, they are complementary tools targeting different pathological dimensions. In ACS and acute thromboembolic complications, SII offers irreplaceable specificity; in chronic coronary artery disease and long-term prognosis, SIRI may provide a more stable indicator of cumulative inflammation; and PIV offers a more comprehensive assessment of immune status. It should be noted that direct head-to-head studies comparing SII, SIRI, and PIV remain limited at present, and the above conclusions require further validation through additional large-scale prospective studies.

### 5.4 Synergistic value with clinical risk models: incremental contribution of an independent dimension

The SII is not intended to replace existing risk assessment tools, but rather to establish a multidimensional risk stratification system by introducing the independent dimension of “thrombotic-inflammatory burden.”

Studies have shown that combining SII with the Global Registry of Acute Coronary Events (GRACE) score significantly improves the prediction of long-term outcomes in ACS (104); combining it with the CHA₂DS₂-VASc score optimizes the assessment of CIN risk (73); synergizing with NT-proBNP produces an amplifying effect, further improving the accuracy of MACE prediction in STEMI patients (71, 105); and combining it with hs-CRP enhances the ability to distinguish CIN (72).

This synergistic effect demonstrates that SII provides a unique dimension of information—the thrombotic-inflammatory interaction status—that goes beyond traditional clinical features (GRACE, CHA₂DS₂-VASc scores), myocardial injury markers (NT-proBNP), and classic inflammatory markers (hs-CRP). By integrating the three-dimensional framework of “clinical features, myocardial injury, and thrombo-inflammatory status,” SII helps establish a more precise, personalized risk assessment framework, providing evidence-based support for enhanced anticoagulant or anti-inflammatory interventions.

## 6 Conclusions and outlook

By integrating platelets (pro-platelet), neutrophils (pro-inflammatory), and lymphocytes (immunoregulatory), SII establishes a comprehensive biomarker for quantifying imbalances in the thrombotic-inflammatory loop. Its computational framework accurately reflects the core pathology of congenital heart disease: platelet-neutrophil synergy drives thrombotic and pro-inflammatory responses, while lymphopenia indicates immune regulatory failure. Compared to single acute-phase proteins [CRP, Interleukin-6 (IL-6)] or dual ratio indices (NLR, PLR), SII more comprehensively captures the complex interactions between thrombosis and inflammation, demonstrating unique value for risk stratification and dynamic monitoring. The predictive performance of SII exhibits marked phenotype-dependence: it peaks in acute coronary syndrome and thrombotic complications, accurately reflecting the intensity of acute thrombo-inflammatory storms; in stable lesions, it continuously monitors chronic low-grade inflammatory burden. This dual role of “acute-phase warning and chronic-phase monitoring” provides a theoretical foundation for clinical stratification applications.

Current evidence supports the use of SII as a phase-specific risk stratification tool, with its application focused on acute thromboinflammatory scenarios: (1) Acute-phase risk warning: In patients with ACS and those at high risk for thrombotic events (LVT, ST, NRP), an SII > 900–1,400 indicates activation of an acute thromboinflammatory storm, warranting consideration of intensified anticoagulation or mechanical protection; A low threshold of 450–650 can be used for highly sensitive screening of complications such as NRP. In the chronic phase of CCS, an SII >650 merely suggests the possibility of anatomical complexity and should not be used as an independent prognostic indicator. (2) Dynamic prognostic monitoring: Dynamic changes in SII have greater prognostic value than single-point measurements; they show a dose-dependent association with short-term MACE, heart failure, and microcirculatory complications, and can provide incremental information for traditional models such as GRACE; however, their predictive efficacy for long-term hard endpoints (>1 year) is limited (AUC 0.59–0.63). (3) Pathophysiological monitoring: As a dynamic indicator of thromboinflammatory activity, it has the potential to monitor the modulatory effects of antiplatelet and anti-inflammatory therapies on thromboinflammatory pathways. All of the above applications are based on observational studies; SII should currently be positioned as a risk stratification tool rather than a basis for treatment decisions.

Limitations of the current evidence include:

1. Study design: Most current studies are retrospective and single-center in design, making it difficult to completely avoid inherent bias. Meta-analyses indicate that the quality of evidence supporting the predictive value of SII is rated as “low” to “very low” (35); effect estimates may be overestimated, and causal inferences should be interpreted with caution.
2. Significant heterogeneity across studies: There is significant heterogeneity among studies regarding the population (from SAP to STEMI), cutoff values (528–2,174), outcome definitions (differences in MACE components), and treatment settings (differences in antiplatelet and statin use), which limits the integration of evidence and standardization of clinical application. Notably, even in the general population, SII levels exhibit age- and sex-dependent patterns-a cohort study involving 8,711 participants showed that SII levels increase with age and are higher in women than in men (mean 459, 95% confidence interval 189–1,168). These inherent population differences further exacerbate the heterogeneity in baseline levels across different study populations (106).
3. Comparisons with next-generation biomarkers remain controversial: Although head-to-head comparative studies between SII and traditional biomarkers such as CRP, NLR, and PLR are relatively well-established, the results of comparisons with next-generation composite indices such as SIRI and PIV show heterogeneity—some studies indicate that SII performs better in acute thrombotic events, while others suggest that SIRI is superior in the assessment of chronic lesions. This inconsistency may stem from differences in the pathological stage, treatment context, and definition of endpoints across studies; further standardization of com

Future research should focus on the following areas: (1) Elucidation of mechanisms: specifically, validation of the thrombus-inflammation pathway. The direct association between SII and *in situ* thrombotic-inflammatory activity remains speculative at this stage. Future research should validate the quantitative association between SII and direct markers of thrombotic inflammation (such as NET degradation products and platelet-neutrophil aggregates) to demonstrate that SII serves as a systemic spillover signal of local thrombotic storms rather than a mere reflection of systemic inflammation. (2) Model optimization: Develop a phenotype-adaptive multidimensional inflammatory risk model. Utilize machine learning to integrate SII with biomarkers such as hs-CRP, IL-6, and SIRI to construct stratified risk models for different coronary artery disease phenotypes, and establish a stratified cutoff value system to address heterogeneity issues. (3) Intervention Validation: Conduct SII-guided randomized controlled trials, implementing intensified antiplatelet therapy in the SII >900 cohort to verify whether high-risk SII individuals benefit from an intensified antiplatelet strategy; additionally, validate whether dynamic SII monitoring can guide the initiation and discontinuation of anti-inflammatory drugs (such as colchicine), thereby facilitating the transition from risk prediction to precision therapy.

By quantifying the overall imbalance of the thrombotic-inflammatory loop, SII provides an integrated biomarker framework for understanding the mechanisms of CHD and for risk stratification. Its predictive performance is phenotype-dependent, being particularly prominent in ACS and thrombotic complications, which aligns with the key driving role of the thrombotic-inflammatory storm; however, its value is limited in chronic lesions, suggesting that clinical application should focus on risk assessment for acute thrombotic events. Due to the heterogeneity and methodological limitations of existing studies, the clinical translation of SII must progress from observational associations to interventional validation to provide evidence-based support for precision anti-inflammatory therapy.
